# Rare Complication of Distal Radial Artery Puncture: Arteriovenous Fistula Formation Following Percutaneous Coronary Intervention

**DOI:** 10.7759/cureus.79541

**Published:** 2025-02-24

**Authors:** Daisuke Yamazaki, Shin Makabe, Mitsunori Yuzurihara

**Affiliations:** 1 Cardiology, Akita Cerebrospinal and Cardiovascular Center, Akita, JPN; 2 Cardiology, Tokyo Kamata Hospital, Kamata, JPN

**Keywords:** arterio-venous fistula, distal radial artery approach, pci (percutaneous coronary intervention), transradial approach, vascular ligation

## Abstract

Distal radial artery puncture is increasingly utilized in percutaneous coronary intervention (PCI) due to its reduced complication rates compared to conventional approaches. However, arteriovenous fistula (AVF) formation, though rare, remains a potential complication.

A 71-year-old man underwent PCI for effort angina of the left main trunk via a distal radial artery (RA) approach. Three years later, he underwent cardiac catheterization and lower limb arteriography for peripheral arterial disease with intermittent claudication. When palpation of the left RA was confirmed, an enlarged cephalic vein with thrill was observed. Ultrasonography revealed an arteriovenous fistula (AVF) with a diameter of 2.0 mm, and arteriography was performed during catheterization to diagnose AVF. Three years after the PCI, the distended cephalic vein was strongly distended, so vascular ligation was performed at another institution. Although a residual, unidentifiable arteriovenous shunt and a weak thrill persisted, the distended cephalic vein showed improvement, and the patient's condition remained favorable. Early detection of distal radial AVFs, potentially manageable by compression, underscores the importance of post-procedural vigilance in PCI patients.

## Introduction

Distal radial artery (RA) puncture has gained popularity in percutaneous coronary intervention (PCI) due to reduced bleeding and vascular complications compared to the femoral approach [1]. Despite these advantages, rare complications such as arteriovenous fistula (AVF) formation can occur. AVFs are abnormal connections between arteries and veins that may develop following arterial puncture, often presenting with a palpable thrill or venous distension. While most AVFs are diagnosed shortly after the procedure [2-4], those associated with distal RA puncture may remain undetected for years due to low shunt flow and minimal symptoms. Here, we report a rare case of AVF three years after distal RA puncture, highlighting the importance of early detection and the potential for minimally invasive management.

## Case presentation

**Figure 1:**
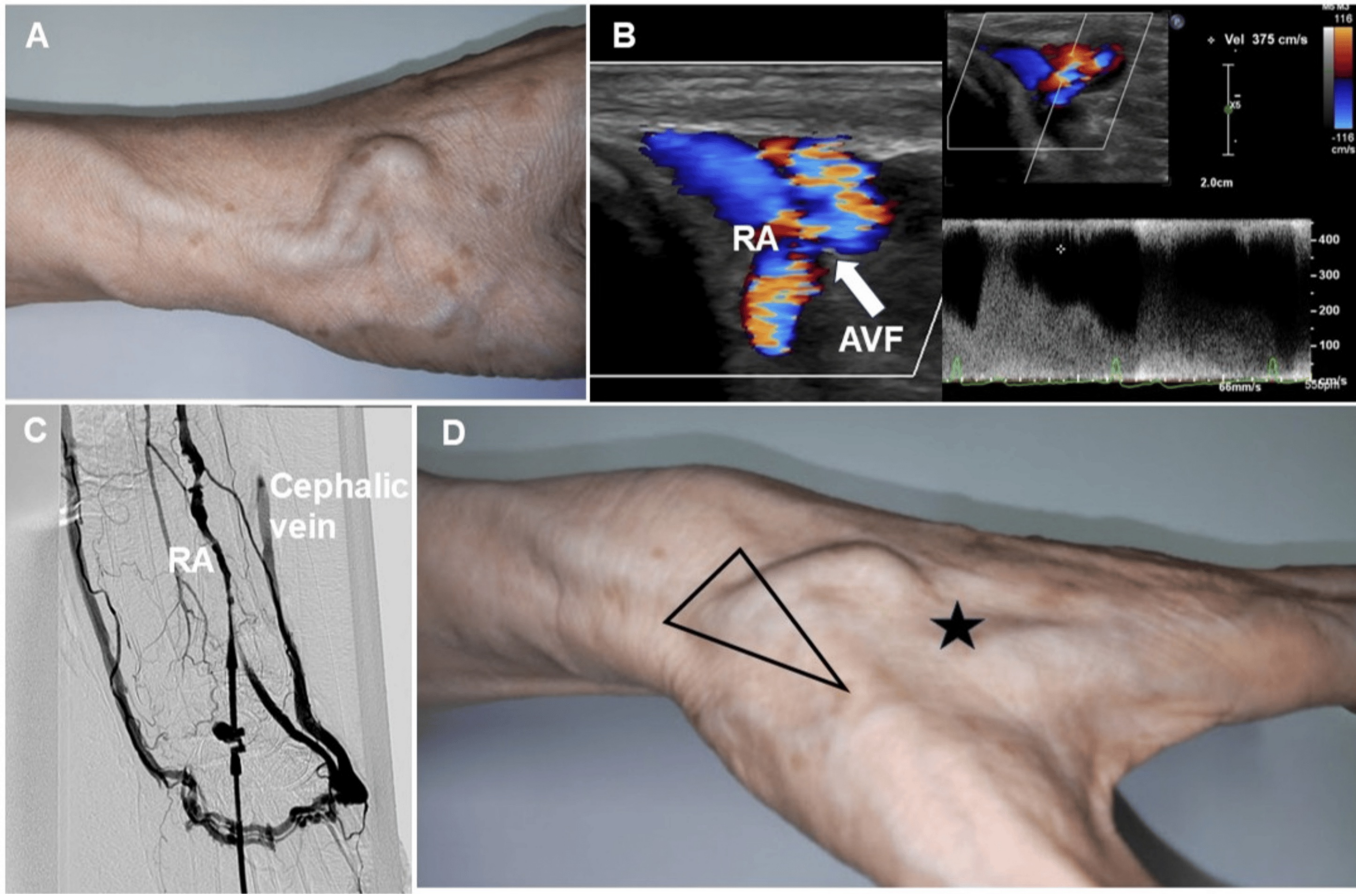
Photographs, echographic findings, and angiogram of arteriovenous fistula formed by distal radial artery puncture. RA: radial artery; AVF: arteriovenous fistula (A) A distended cephalic vein is observed. (B) The arrow shows the entrance to the arteriovenous fistula, where the radial artery connects to the vein. A superficial ultrasonography revealed an arteriovenous fistula in the distal radial artery with a diameter of 2.0 mm and a flow velocity of 3.75 m/sec. (C) Angiography from the radial artery. A shunt was revealed between the distal RA and the dorsal venous network of the hand, and the basilic and cephalic veins were contrasted. It communicates from the radial artery to the vein near the metacarpal bone of the thumb. (D) Common distal radial artery puncture sites (triangle) and the puncture site (star) in this case.

The case was a 71-year-old man who had undergone PCI three years previously for in-stent restenosis of the left main trunk due to effort angina. At that time, a Glidesheeth Slender ^Ⓡ^6Fr (TERUMO, Tokyo, Japan) was inserted via a left distal RA approach under ultrasound guidance. Glidesheeth Slender ^Ⓡ^ is a thinner introducer sheath designed to reduce vascular complications. Three years later, he underwent coronary angiography and lower limb angiography for follow-up after PCI for peripheral arterial disease with intermittent claudication. Before the examination, we checked the RA and were able to feel the thrill. In addition, we observed the distended cephalic vein as shown in Figure 1A.

Surface ultrasonography revealed an arteriovenous fistula (AVF) at the puncture site. The maximum flow velocity at the shunt was 3.75 m/s and the diameter of the fistula was 2.0 mm (Figure 1B, Video 1).

**Video 1:** Echo findings of the arteriovenous fistula. The arrow shows the entrance to the arteriovenous fistula, where the radial artery connects to the vein.

Cardiac catheterization was performed by puncturing the conventional left RA. A 21-gauge cannula was inserted, and contrast was injected into the left RA prior to sheath insertion. A shunt was revealed between the distal RA and the dorsal venous network of the hand, and the basilic and cephalic veins were contrasted (Figure 1C, Video 2).

**Video 2:** Radial artery angiography. A shunt was revealed between the distal RA and the dorsal venous network of the hand, and the basilic and cephalic veins were contrasted. It communicates from the radial artery to the vein near the metacarpal bone of the thumb.

Although there was no evidence of congestive heart failure due to left-to-right shunting, the cephalic vein was severely distended and a strong thrill could be felt, so vascular ligation was performed at another institution. A skin incision was made at the anatomical snuffbox, a common puncture site for the distal radial artery; however, the AVF involving the radial artery was not identified. Furthermore, when the distended cephalic vein was traced through an incision in the more distal part, the shunt vein was identified and was palpable with a strong thrill. As it was not possible to identify the fistula with the RA for the deep part, the shunt vein was ligated and cut with 3-0 silk thread, and the distension of the cephalic vein disappeared. The strong thrill disappeared, but a mild thrill still remained in the deep area. However, it was difficult to identify the deep area, so the wound was closed, and the procedure was completed. After the surgery, the mild thrill remained, but the distension of the cephalic vein disappeared, and the patient's condition remained stable with no signs of heart failure.

## Discussion

This case highlights a rare complication of distal radial artery puncture, with the AVF diagnosed three years post-procedure. Distal RA puncture is a method of puncturing the RA more distally than the conventional RA that runs at the level of the wrist and was first reported in 2017 [1]. By puncturing a smaller diameter peripheral artery than the conventional RA, it is expected to be less invasive and have fewer complications. The distal RA puncture approach generally involves puncturing the base of the anatomical snuffbox. However, in this case, the puncture was performed under ultrasound guidance and was made near the intersection of the more peripheral extensor pollicis longs tendon and the second metacarpal base (Figure 1D). It is thought that the AVF was formed because the superficial palmar branch of the RA was punctured after penetrating the dorsal venous network of the hand.

Table 1 summarizes recent case reports of the AVF formed by RA puncture [2-10]. In all cases, the diagnosis was easily made using a minimally invasive ultrasound examination. Only one case showed signs of heart failure due to a left to right shunt, and the others were only partially symptomatic around the puncture site. In all but two cases, AVFs were found more than one month after puncture, and in the present case, distension of the cephalic vein was noted three years after distal RA puncture. The RA puncture is likely to have the paradoxical aspect that the discovery of AVFs may be delayed due to its minimally invasive nature. All cases in which AVFs were found after a period of time, required surgical treatment. In most cases, the diagnosis is made by being aware of or noting a thrill at the puncture site. Interestingly, there were cases where the patients themselves were aware of the beating thrill, but they just observed it [7]. If they had been instructed to check the puncture site for abnormalities, they would have been able to diagnose the AVF much earlier.

**Table 1:** Summary of recent cases in which an arteriovenous fistula was formed by radial artery puncture. PCI: percutaneous coronary intervention; CAG: coronary angiography; AVF: arteriovenous fistula; US: ultrasound; CT: computed tomography

Author	Year	Age	Sex	Symptoms	Post PCI or CAG AVF diagnosis	Means of diagnosis	Intervention	Outcome
Mehta et al. [2]	2020	74	M	Thrill, numbness	1 day	Ultrasound	Conservative	Good
Herzallah et al. [3]	2021	85	M	Despnea, wrist pain	2 months	Ultrasound	Surgical ablation	Good
Htun et al. [4]	2022	61	F	Thrill	3 days	Ultrasound	Compression	Good
Gu et al. [5]	2022	73	F	Thrill, edema, fingers numbness	11 months	Ultrasound, computed tomography	Ligation	Good
Maeba et al. [6]	2022	71	M	Wrist pain, cold sensation	3 months	Ultrasound, computed tomography	Ligation	Good
Okam et al. [7]	2023	51	F	Thrill, paresthesia	6 months	Ultrasound	Ligation	NA
Okam et al. [7]	2023	72	F	Thrill	2 years	Ultrasound	Ligation	Good
Dakik et al. [8]	2023	56	M	Mass	6 weeks	Ultrasound	Surgery	Good
Mahanta et al. [9]	2024	45	M	Thrill, warm sensation	6 months	Ultrasound	Ligation	Good
Senger et al. [10]	2024	44	F	Thrill, edema, wrist pain	7 weeks	Ultrasound	Ligation	Good

Because the AVF was formed when the catheter sheath was inserted, the shunt blood flow was low, unlike the shunt formed during dialysis, and it is thought that the angulation of the cephalic vein took time to occur. The diameter of the AVF in this case was only about 2 mm and the shunt blood flow was less than that of a hemodialysis shunt. Perhaps because most cases are diagnosed after a period of time has passed, most are cured by surgical ligation rather than compression. There was a case report by Htun et al in 2022 of a patient who had the AVF formed by puncturing the distal RA [4].

This case report differed from the other cases in that the AVF was diagnosed early, three days after PCI, and the fistula was successfully closed by compression. The AVF formed by the distal RA puncture is peripheral, so the shunt blood flow is less than that of the AVF formed by the conventional RA puncture, and it is thought that it was able to be healed by compression because it was detected early. Although further data is needed because there are few case reports of the AVF formed by distal RA puncture, it is hoped that the shunt blood flow will be less than that of conventional AVFs, so if it can be detected early, the possibility of healing by compression may increase. In recent years, PCI has also become less invasive, and patients are often discharged soon after a procedure. For this reason, there are few opportunities to discover AVF during a hospital stay. By instructing patients to check for a thrill at the puncture site, it is possible to detect the AVFs early after discharge and close them by compression.

## Conclusions

Early detection of AVFs following distal radial artery puncture is essential to prevent progressive venous distension and facilitate minimally invasive management. This case underscores the importance of post-procedural vigilance and patient education in identifying complications of distal RA access. By emphasizing proactive monitoring and education, complications associated with distal radial artery access can be effectively managed, ultimately improving patient safety and preserving vascular integrity.
